# Supplementary material for: Novel Optical Methodology Unveils the Impact of a Polymeric Pour-Point Depressant on the Phase Morphology of Waxy Crude Oils
Source: Polymers (Basel). 2024 Jul 6;16(13):1933. doi: 10.3390/polym16131933 (PMC11243897; doi:10.3390/polym16131933)
Supplement: Supplementary file 1 [file polymers-16-01933-s001.zip › polymers-3050077-supplementary.pdf]

polymers

MDPI

Article

Novel Optical Methodology Unveils the Impact of a Polymeric Pour-Point Depressant on the Phase Morphology of Waxy Crude Oils

Irene Perna [1], Rosalia Ferraro [1,2], Consiglia Carillo [1], Salvatore Coppola [3,*] and Sergio Caserta [1,2,*]

[1] Department of Chemical, Materials and Production Engineering, University of Naples Federico II, P. le V. Tecchio 80, 80125 Naples, Italy; irene.perna@unina.it (I.P.); rosalia.ferraro@unina.it (R.F.); consiglia.carillo@libero.it (C.C.)

[2] CEINGE Advanced Biotechnologies Franco Salvatore, Via G. Salvatore 436, 80131 Naples, Italy

[3] Elastomers Research and Development Centre, Versalis S.p.A. (Eni), I-48100 Ravenna, Italy

* Correspondence: salvatore.coppola@versalis.eni.com (S.C.); sergio.caserta@unina.it (S.C.)

Supplementary materials

Temperature sweep tests were conducted to analyse the evolution of G'' against the temperature for each sample of the crude oils investigated in this study, also highlighting the effect of paraffin concentration (samples B, C, and D), extraction time (samples E and F), and PPD (samples E, G, and H). Triangle down symbols represent cooling, while triangle up symbols represent heating, distinguished by fill and empty symbols for the first and second cycle, respectively. The imposed thermal profile follows the previously defined code. For all samples, G'' decreases significantly with increasing temperature, dropping by at least four orders of magnitude. For instance, in the case of sample D, G'' rises from 10^0 to 10^5 as the temperature drops from 30°C to 4°C.

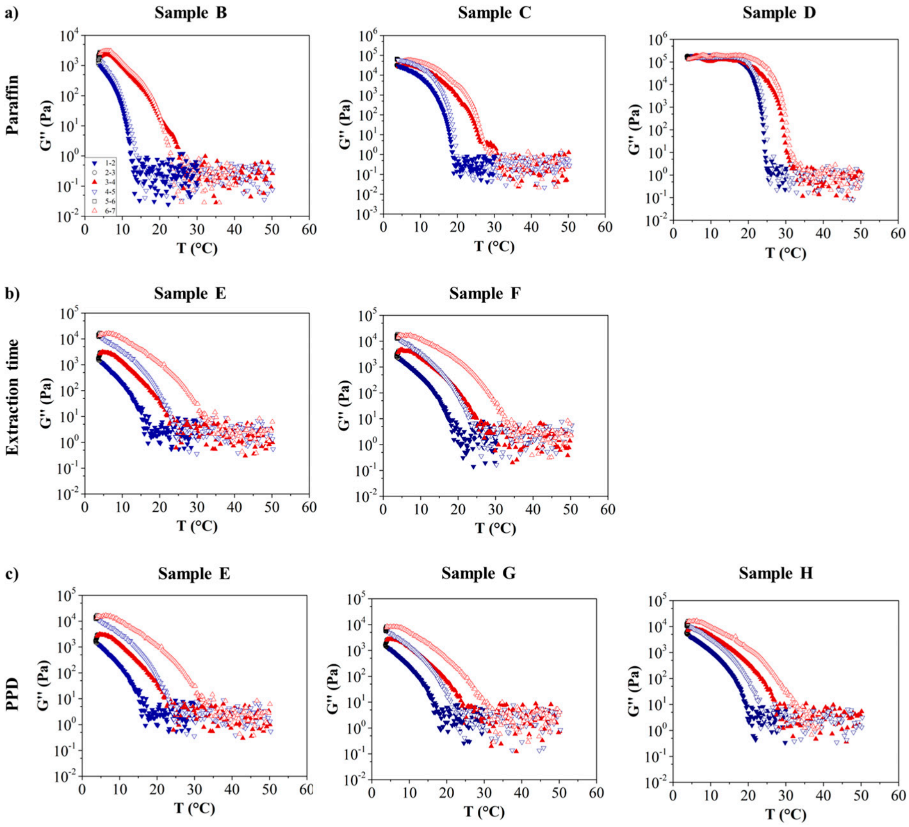

Citation: Perna, I.; Ferraro, R.; Carillo, C.; Coppola, S.; Caserta, S. Novel Optical Methodology Unveils the Impact of a Polymeric Pour-Point Depressant on the Phase Morphology of Waxy Crude Oils. *Polymers* **2024**, *16*, 1933. https://doi.org/10.3390/polym16131933

Academic Editor: Marcelo Antunes

Received: 24 May 2024
Revised: 27 June 2024
Accepted: 3 July 2024
Published: 6 July 2024

Copyright: © 2024 by the authors. Submitted for possible open access publication under the terms and conditions of the Creative Commons Attribution (CC BY) license (https://creativecommons.org/licenses/by/4.0/).

Figure S1. Viscous modulus, G'', *versus* temperature, T, highlighting the effect of paraffin concentration (**a**) for sample B, C, and D, extraction time (**b**) for sample E, and F and PPD percentage (**c**) for sample E, F, and H. The imposed thermal profile follows the previously defined code: points 1-2 for the first cooling ramp, points 2-3 for a 10-minute holding period at a specified temperature, points 3-4 for the first heating ramp, points 4-5 for the second cooling ramp, points 5-6 for another 10-minute holding period at 4°C, and points 6-7 for the second heating ramp. Triangle down blue symbols represent cooling, while triangle up red symbols represent heating, distinguished by fill and empty symbols for the first and second cycle, respectively.

Temperature sweep tests were conducted to analyze the evolution of η* against the temperature for each sample of the crude oils investigated in this study, also highlighting the effect of paraffin concentration (samples B, C, and D), extraction time (samples E and F), and PPD (samples E, G, and H). Triangle down symbols represent cooling, while triangle up symbols represent heating, distinguished by fill and empty symbols for the first and second cycle, respectively. The imposed thermal profile follows the previously defined code. For all samples, η* decreases significantly with temperature by at least four orders of magnitude. Indeed, the viscosity of sample C increases from 10^{-1} to 10^{4} as the temperature decreases during the first cooling ramp.

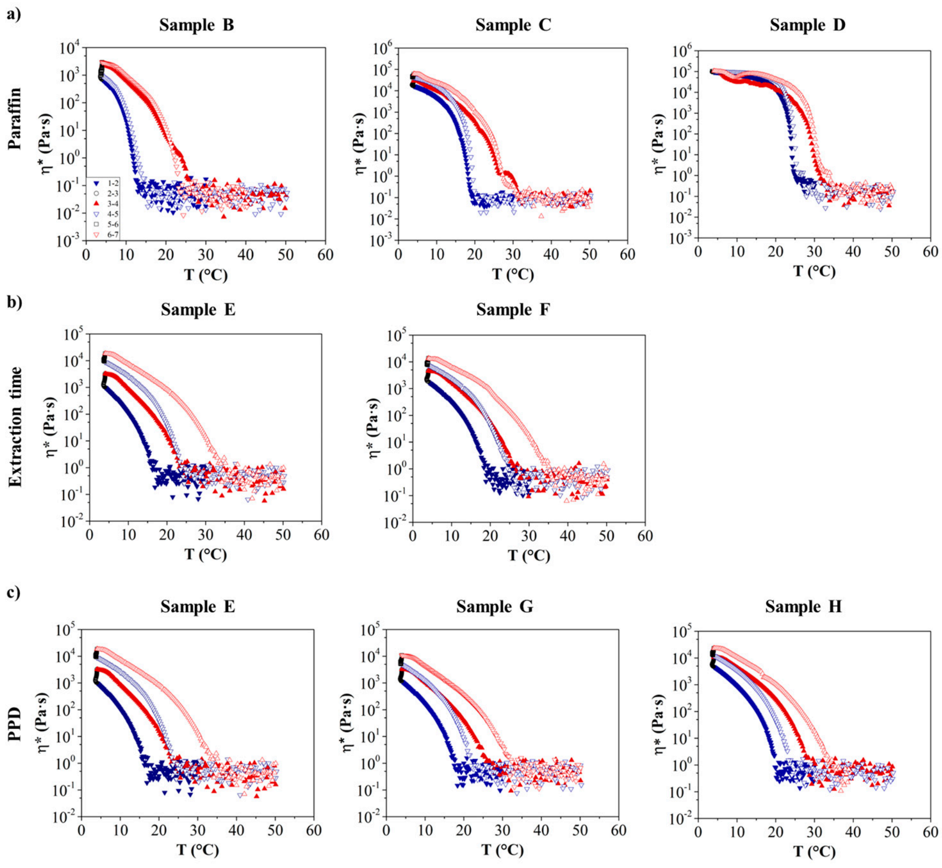

Figure S2. Complex viscosity, η*, versus temperature, T, highlighting the effect of paraffin concentration (**a**) for sample B, C, and D, extraction time (**b**) for sample E, and F and PPD percentage (**c**) for sample E, F, and H. The imposed thermal profile follows the previously defined code: points 1-2 for the first cooling ramp, points 2-3 for a 10-minute holding period at a specified temperature, points 3-4 for the first heating ramp, points 4-5 for the second cooling ramp, points 5-6 for another 10-minute holding period at 4°C, and points 6-7 for the second heating ramp. Triangle down blue symbols represent cooling, while triangle up red symbols represent heating, distinguished by fill and empty symbols for the first and second cycle, respectively.

Heat Flow [W/g] is plotted against temperature [°C] to generate the Cumulative Heat Flow curve. The cooling phase is illustrated by the blue lines, whereas the heating phase is denoted by the red lines. Arrows with corresponding color codes are included to aid in visualizing the thermal cycle.

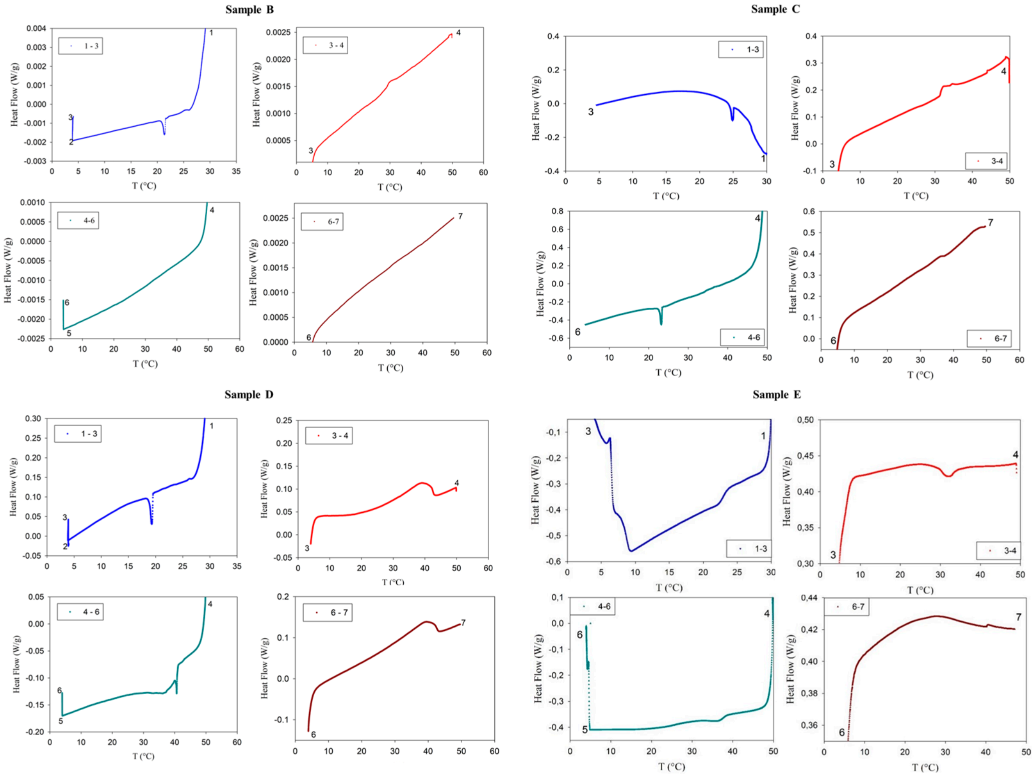

Figure S3. Heat Flow *versus* temperature, T, effect for samples B, C, D and E. The imposed thermal profile follows the previously defined code: points 1-2 for the first cooling ramp, points 2-3 for a 10-minute holding period at a specified temperature, points 3-4 for the first heating ramp, points 4-5 for the second cooling ramp, points 5-6 for another 10-minute holding period at 4°C, and points 6-7 for the second heating ramp.

Wax Appearance Temperature (WAT) of both first and second cycle has been determined for all the sample and for each experiment performed. As an example, the WAT evaluation for the second cycle is reported for sample D. In the case of DSC (**Figure S4a**), the WAT is determined by identifying the temperature at which a decrease in the CHF is observed while decreasing the temperature (from right to left). Consequently, the WAT is identified as the intercept between two lines with different slopes (highlighted by the yellow dotted line in the plot). Similarly, in the rheology measurements (**Figure S4b**), the WAT is evaluated as the temperature at which an increase in the storage modulus (G') occurs. In CPM (**Figure S4c**), the WAT can be established by pinpointing the temperature at which the relative intensity (I/I_0) increases during the cooling process.

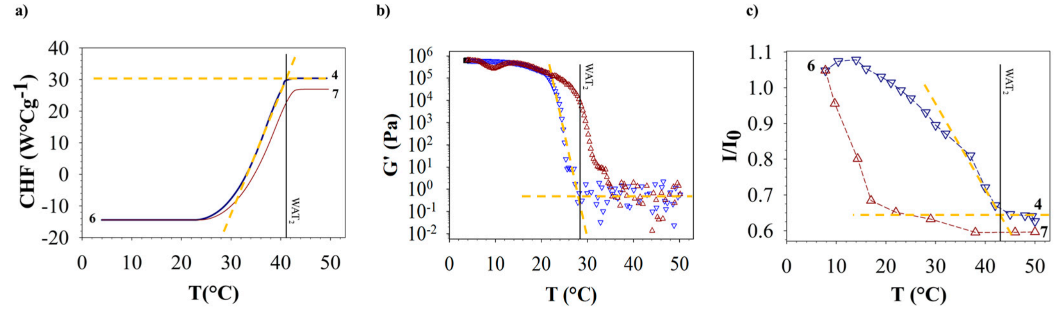

Figure S4. Determination of WAT_2 of sample D for second cycles for all the methodologies: DSC (**a**), rheology (**b)** and CPM (**c**).
